# Demographic and Temporal Trends in Thyroid Cancer Incidence in the United States From 1999 to 2020: An Analysis of Age, Gender, and Race

**DOI:** 10.7759/cureus.84305

**Published:** 2025-05-17

**Authors:** Daaniya Tariq, Onuralp Kafali, Jorge Cabrera Macias, Nithin Karnan, Min Ji Kim

**Affiliations:** 1 Department of Internal Medicine, Shaheed Suhrawardy Medical College and Hospital, Dhaka, BGD; 2 Department of Internal Medicine, Duzce University Medical School, Duzce, TUR; 3 School of Medicine, University of Guayaquil, Guayaquil, ECU; 4 Department of Internal Medicine, K.A.P. Viswanatham Government Medical College, Tiruchirappalli, IND; 5 Department of Internal Medicine, Tashkent Pediatric Medical Institute, Tashkent, UZB

**Keywords:** age, cdc-wonder, crude incidence rates, demographic trends, epidemiology, gender, public health, race, thyroid cancer, united states

## Abstract

Purpose: Thyroid cancer is a growing concern in public health and has noticeable disparities in incidence among demographic subgroups and regions. This study analyzed the incidence of thyroid cancer from 1999 to 2020 using the Centers for Disease Control and Prevention Wide-ranging Online Data for Epidemiologic Research (CDC-WONDER) database, focusing on age, race, and gender. Additionally, the study sought to identify geographical disparities in thyroid cancer incidence and understand temporal trends over the study period.

Methods: The study extracted thyroid cancer incidences from the CDC-WONDER database from 1999 to 2020 by age, race, and gender demographics. Crude incidence rates were calculated based on a 100,000 population, and temporal trends were identified to peak incidence periods, using Excel (Microsoft Corporation, Redmond, WA) and RStudio version 4.3.1 (Posit Software, Boston, MA).

Results: The data included a total population of 6,722,531,004, with 837,105 (0.0124%) diagnosed cases of thyroid cancer between 1999 and 2020. The crude rate was highest in the 65-74-year age group with a rate of 22.5 per 100,000, in females at 18.4 per 100,000, and in the White race with a rate of 13.2 per 100,000, while California had the highest statewise distribution, exceeding 75,000 per 100,000 population. From temporal trends, there is a general increasing trend in the incidence of thyroid cancer, with the crude incidence rate for the period under study amounting to 12.5 per 100,000 population, and a peak observed in 2015.

Conclusion: The findings point to significant demographic and geographic disparities in the incidence of thyroid cancer across the United States. Such an insight should be crucial for designing directed public health interventions and well-conceived screening protocols for high-risk subgroups. Geographic disparities further aid in resource mobilization and health service planning. This study emphasizes the importance of good cancer surveillance and continuous monitoring of public health strategies, which would provide early detection to attain better patient outcomes.

## Introduction

Cancer is a complex and pervasive health issue characterized by abnormal cell growth, posing significant challenges to both patients and healthcare systems worldwide. Annually, millions of individuals succumb to cancer, highlighting its profound impact on global health. In the United States, cancer claims the lives of over 600,000 people each year [1]. Beyond its physical toll, cancer carries profound emotional and socioeconomic burdens. Among the diverse spectrum of cancers, thyroid cancer has recently gained prominence due to its shifting epidemiological landscape within the United States.

The study of thyroid cancer incidence is crucial for understanding its epidemiology and identifying contributing factors to its prevalence [2]. Such research not only reveals demographic trends but also informs the development of effective public health policies and clinical strategies. By examining temporal patterns and demographic variations in thyroid cancer incidence, researchers can pinpoint geographic disparities, assess risk factors, and evaluate the impact of genetic and environmental influences. This knowledge is essential for guiding resource allocation, improving screening protocols, and tailoring prevention and treatment approaches.

Despite increasing interest in thyroid cancer epidemiology, there remains a noticeable gap in comprehensive studies examining its incidence and demographic variations across the United States. Studies by Davies and Welch [3] and Lim et al. [4] emphasize the complexities underlying these trends, attributing them to advancements in detection methods and potential changes in disease prevalence over time. Historical perspectives from Zimmerman et al. provide additional context, illustrating long-term patterns and underscoring the ongoing need for robust surveillance and research [5].

Utilizing authoritative sources such as the Centers for Disease Control and Prevention Wide-ranging Online Data for Epidemiologic Research (CDC-WONDER) database ensures the reliability of the data. These efforts are critical for advancing our understanding of thyroid cancer trends and informing evidence-based strategies for cancer surveillance, prevention, and management in the United States.

Aims and objectives

The objective of the study is to analyze the incidence of thyroid cancer in the United States from 1999 to 2020 using the CDC-WONDER database, based on demographic variables of age, gender, and race. Additionally, the study sought to identify geographical disparities in thyroid cancer incidence and understand temporal trends over the study period.

## Materials and methods

Study design and setting

A retrospective observational research study was conducted using the CDC-WONDER database. Data were extracted on May 18, 2024, with the combined contribution of the coauthors. This study involves nonhuman participant research, as the CDC-WONDER database contains deidentified, publicly available data. Therefore, no ethics committee approval was required for this study.

Data collection

Data collection was performed on a single day, on May 18, 2024, focusing on cancer statistics, specifically cancer incidence from 1999 to 2020. The inclusion criteria for the study involved selecting data specific to thyroid cancer sites and focusing on temporal trends and demographic variables. For temporal trends, the variable “year” was included. For demographic analysis, variables such as age, gender, and race were selected.

The demographic variables selected included age groups, gender, and race. The age groups analyzed were <15, 15-24, 25-34, 35-44, 45-54, 55-64, 65-74, and ≥75 years. Data were collected for both male and female patients, and the racial categories included were American Indian or Alaskan Native, Asian or Pacific Islander, Black or African American, and White. Other cancers that did not meet these criteria were excluded from the analysis.

Data analysis

The extracted data were exported to a Microsoft Excel spreadsheet for initial organization and preliminary analysis. RStudio version 4.3.1 (Posit Software, Boston, MA) was utilized for visualizations, including figures and graphs, created using the GGPlot2 package, version 3.5.0 (Posit Software).

## Results

From 1999 to 2020, the incidence of thyroid cancer was 837,105, in 6,722,531,044 of the total population. The crude rate per 100,000 was 12.5. Table 1 shows the demographic characteristics of thyroid cancer patients in the United States from 1999 to 2020 based on age, gender, and race. The highest crude rate was observed among the 65-74-year age group, with a rate of 22.5 per 100,000. Based on gender, the highest crude rate was observed in women (18.4 per 100,000), and based on race, the highest crude rate was observed among the White race (13.2 per 100,000).

**Table 1:** Demographic characteristics of thyroid cancer patients in the United States from 1999 to 2020 based on age, gender, and race

Variables	Population	Count, n (%)	Crude rate per 100,000
Age			
<15 years	1,331,960,119	3,916 (0.47%)	0.3
15-24 years	932,609,300	37,603 (4.49%)	4
25-34 years	917,100,206	106,504 (12.72%)	11.6
35-44 years	927,607,896	160,894 (19.22%)	17.3
45-54 years	924,339,992	187,603 (22.41%)	20.3
55-64 years	763,946,196	164,032 (19.6%)	21.5
65-74 years	508,470,217	114,220 (13.64%)	22.5
≥75 years	416,497,118	62,333 (7.45%)	15
Gender			
Female	3,417,038,782	627,084 (74.91%)	18.4
Male	3,305,492,262	210,021 (25.09%)	6.4
Race			
American Indian or Alaskan Native	88,290,475	5,232 (0.63%)	5.9
Asian or Pacific Islander	373,820,618	48,595 (5.81%)	13.0
Black or African American	914,358,967	63,224 (7.55%)	6.9
White	5,346,060,984	706,426 (84.39%)	13.2
Other races	Not applicable	13,628 (1.63%)	Not applicable

**Figure 1:**
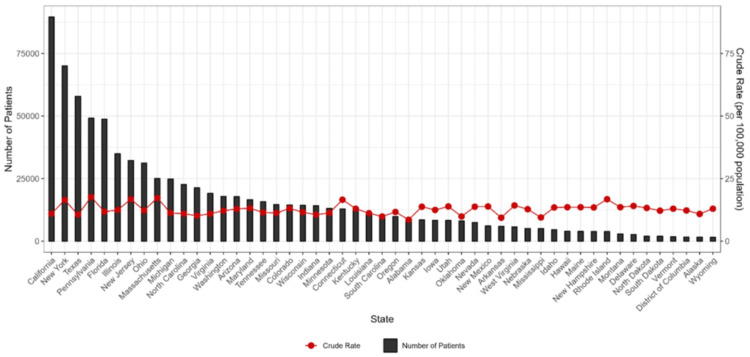
Statewise distribution of thyroid cancer patients in the United States from 1999 to 2020

Figure 1 shows the statewise distribution of thyroid cancer patients in the United States from 1999 to 2020. In the last 22 years, the incidence of thyroid cancer was highest in California, followed by New York and Texas. It was lowest in Wyoming.

**Figure 2:**
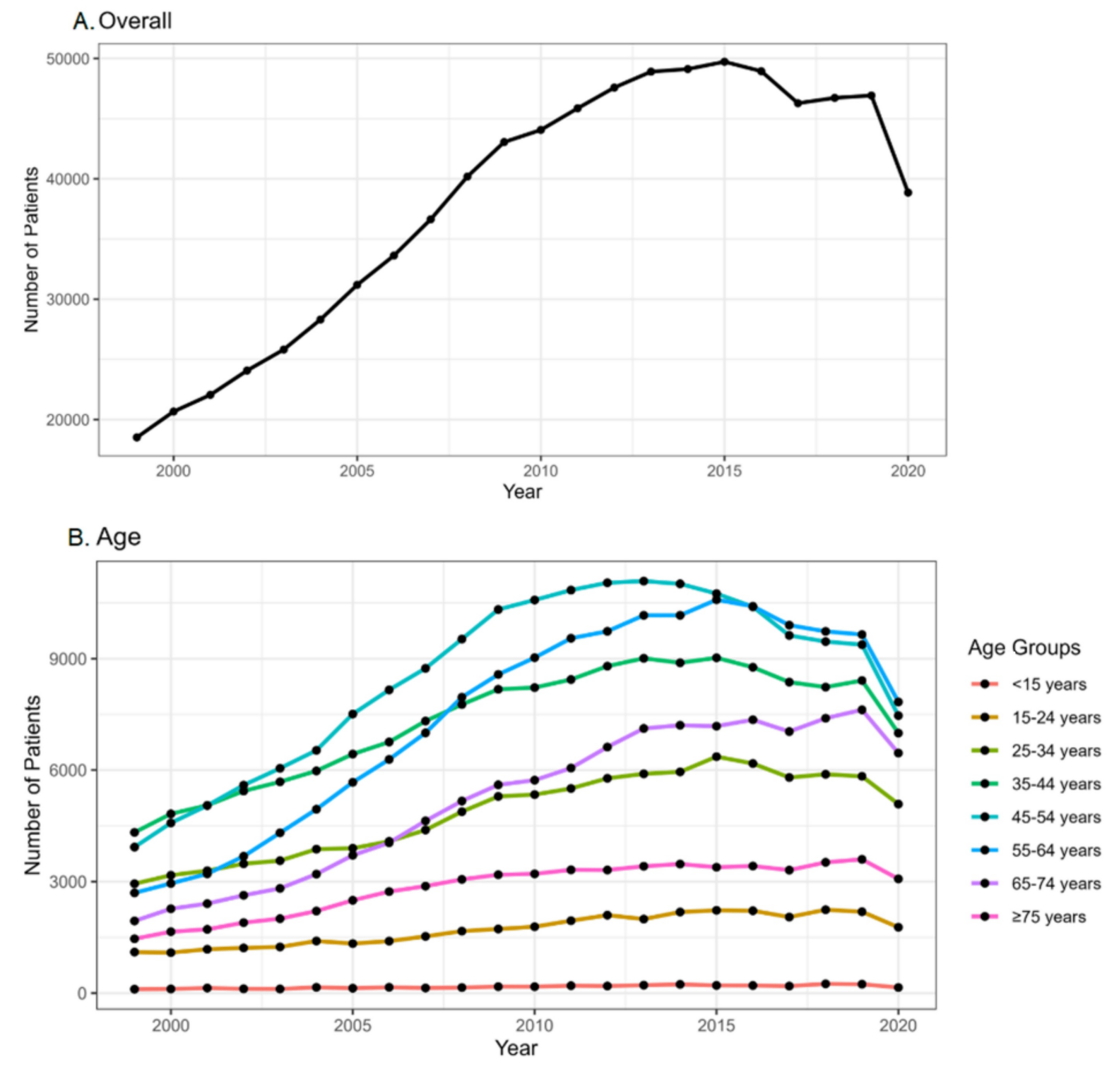
Temporal trends in the incidence of thyroid cancer in United States from 1999 to 2020. (A) Overall temporal trends. (B) Temporal trends based on age

**Figure 3:**
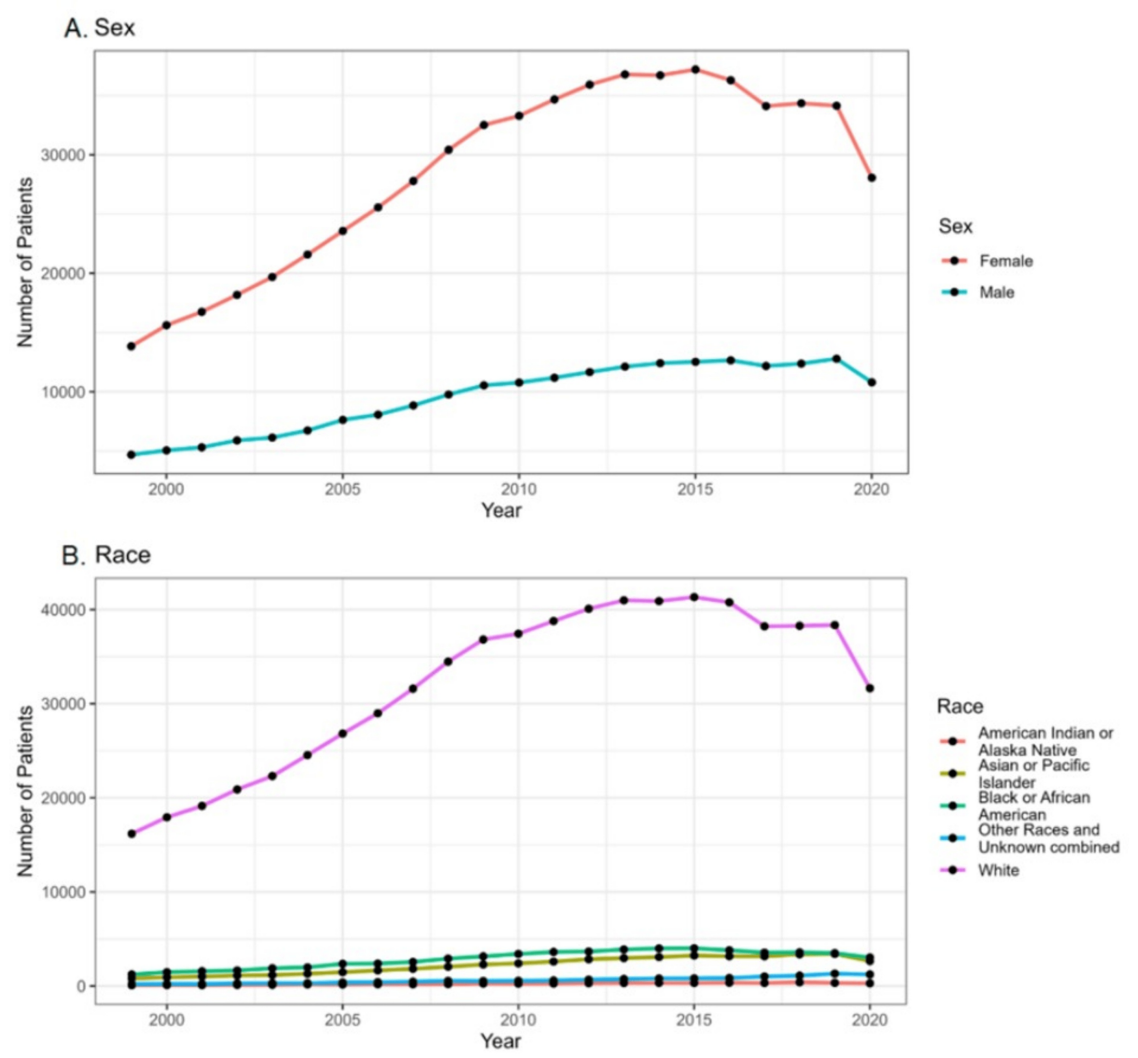
Temporal trends in the incidence of thyroid cancer in United States from 1999 to 2020 based on (A) gender and (B) race

Figures 2, 3 show temporal trends in the incidence of thyroid cancer in the United States from 1999 to 2020 based on age, gender, and race. Overall temporal trends over the years show a steady rise in the annual number of patients diagnosed with thyroid cancer, which peaked during 2015 (Figure 2A). Based on the crude rates per 100,000 population, the incidence appears to be rising in the age group of 45-54 years, where the crude rate is 20.3 per 100,000. The incidence seems to be relatively lower in the age groups of <15 years (0.3 per 100,000) and ≥75 years (15 per 100,000) (Figure 2B).

The data indicate a higher crude rate among women (18.4 per 100,000) compared to men (6.4 per 100,000), suggesting a rising trend in incidence among women. Conversely, men have a lower crude rate, indicating a potentially stable or declining trend in incidence among men (Figure 3A). The data further indicated that the crude rate for thyroid cancer is highest among White racial individuals (13.2 per 100,000) (Figure 3B).

## Discussion

This retrospective study analyzed thyroid cancer incidence trends in the United States over 22 years using data from the CDC-WONDER database. The crude incidence rate was found to be 12.5 per 100,000 people, with the highest incidence observed in the 65-74-year age group, among women, and within the White racial population. These findings are consistent with global trends reported by the International Agency for Research on Cancer, which demonstrated increasing incidence rates of thyroid cancer, particularly in women and in subclinical forms, across 25 countries from 1998 to 2012 [6,7]. Our findings also align with a study from the Surveillance, Epidemiology, and End Results (SEER)-9 database, which found a 3% annual increase in thyroid cancer incidence from 1974 to 2013 and rising mortality in advanced-stage cases [4].

The observed age-specific and sex-specific trends may be attributed to a combination of improved diagnostic modalities, increased health-seeking behavior, and hormonal or environmental influences that may predispose older individuals and females to higher risk. Additionally, increased use of high-resolution imaging such as CT scans has led to a rise in incidentally detected subcentimetric thyroid nodules, as noted in another U.S. cohort study, which reported a sharp increase in overdiagnosis without a corresponding change in mortality [8]. This may explain the rise in crude rates observed in our study, particularly over the last two decades.

Notably, our study found a higher incidence among the White population compared to other racial groups, a trend also seen in SEER-13 data, which reported the highest annual increase in incidence among White individuals (5.6%) compared to Black (4.8%), American Indian (3.2%), and Asian groups (2.3%) [9]. The reasons for this racial disparity could be multifactorial, potentially influenced by access to healthcare, differences in environmental exposures, healthcare utilization patterns, and variations in genetic predisposition. Further compounding this, studies have shown that individuals from high socioeconomic strata with full insurance coverage experience higher incidence rates, possibly due to increased screening and detection, while underinsured individuals see reduced detection rates even within high socioeconomic status (SES) groups [10]. This suggests that healthcare access and socioeconomic factors significantly influence observed incidence rates.

Globally, a positive correlation between high SES and thyroid cancer incidence, coupled with lower mortality in high SES countries, points toward the role of healthcare infrastructure in early detection and management [11].

Environmental factors such as radiation exposure, iodine intake variations, and endocrine-disrupting chemicals have also been implicated in thyroid carcinogenesis. While our dataset does not include such exposures, their potential contribution cannot be overlooked and merits further exploration. Additionally, one ecological study found that countries with higher socioeconomic development had higher incidence but lower mortality from thyroid cancer, again suggesting a significant role for early detection and treatment availability [11].

Moreover, disparities in treatment practices, such as undertreatment or less frequent surgical intervention among Black patients, further highlight the need to address racial and economic inequities in thyroid cancer care [12]. Multivariable logistic regression models were employed to estimate the adjusted association of receipt of appropriate extent of surgery and radioactive iodine, specifically under and over treatment, among different racial groups, concluded that proportions of patients who are receiving thyroid cancer-related surgeries are lower in individuals of the Black race, compared to individuals of the White race [12].

Limitations

This study is limited by the exclusion of data from 2021 to 2023 due to disruptions in data collection during the COVID-19 pandemic. Additionally, details regarding cancer stage, grade, treatment, and outcomes were not available from the dataset and hence could not be evaluated.

## Conclusions

This study found that the crude incidence rate of thyroid cancer in the United States from 1999 to 2020 was 12.5 per 100,000 population, with the highest rates observed among individuals aged 65-74 years (22.5 per 100,000), women (18.4 per 100,000), and those of White race (13.2 per 100,000). While incidence rose steadily over the years, a decline has been noted since its peak in 2015.

These findings highlight notable demographic and geographic disparities in thyroid cancer incidence, underscoring the need for targeted public health interventions and tailored screening strategies for high-risk groups. Recognizing geographic patterns can also facilitate more equitable allocation of healthcare resources and informed decision-making in cancer prevention efforts. Ultimately, this study underscores the importance of robust cancer surveillance systems and continuous evaluation of public health policies to improve early detection and optimize patient outcomes.
